# Acquiring and Maintaining Technical Skills Using Simulation: Initial, Maintenance, Booster, and Refresher Training

**DOI:** 10.7759/cureus.5729

**Published:** 2019-09-23

**Authors:** Anne Sullivan, Summer Elshenawy, Anne Ades, Taylor Sawyer

**Affiliations:** 1 Pediatrics: Neonatology, Boston Children's Hospital, Harvard Medical School, Boston, USA; 2 Pediatrics: Neonatology, The Children's Hospital of Philadelphia, Philadelphia, USA; 3 Pediatrics, Children's Hospital of Philadelphia, Philadelphia, USA; 4 Pediatrics, University of Washington School of Medicine, Seattle, USA

**Keywords:** simulation, skill maintenance, medical education, simulation based medical education, refresher training, booster training, skills training, competency-based medical education

## Abstract

Simulation-based education has been shown to be an effective tool to mitigate skill decay. However, many of the strategies reported in the literature have overlapping terminology with little consensus on the timing of the strategy to prevent skill decay. In this review, we propose and provide a standardized nomenclature and framework for simulation strategies used to obtain, maintain, or regain skills that are decaying. This framework delineates four types of training: initial, maintenance, booster, and refresher. The framework differentiates these training types based on the learner competency at the time of the training, as well as the frequency and intensity of the training. Initial training is aimed at “novice” learners with the goal to achieve competency. Once competency is achieved, maintenance training prevents skill deterioration through low-dose high-frequency (LDHF) training. Booster training is used when the learner is still proficient, but competency begins to wane. Booster training occurs less frequently than maintenance training but with greater intensity to overcome the skill decay that occurs over time. Refresher training is aimed at re-establishing skill levels after competency has reached unsatisfactory levels. Refresher training is higher intensity than booster and maintenance training. We describe simulation-based strategies reported in the literature that can be used for each type of training. We conclude that there should be an increased emphasis in medical education towards maintenance and booster training in order to preserve skills before competency is lost.

## Introduction and background

The goal of medical education is to train competent practitioners with the skills necessary to provide safe and independent patient care. An integral part of the medical education process is the acquisition of the technical and procedural skills necessary for clinical practice. Technical skills are acquired during undergraduate and graduate medical education through simulation and supervised clinical practice. After initial skill acquisition, continued independent clinical practice and experience is necessary to achieve competence. However, once competence is achieved, many skills are rarely used, thus resulting in skill decay [1]. Maintaining an adequate level of competency over time is key to patient safety. Developing strategies to aid in the acquisition and maintenance of technical skills is an area of intense investigation. Several recent reports have focused on the use of simulation-based training to aid in skill acquisition and maintenance. 

Simulation-based education is becoming more prevalent in medical education; it improves clinical skills in a controlled and safe practice environment by providing the opportunity for repetition and consolidation of skill [2]. Simulation-based education has been shown to be an effective tool for initial training, to increase skill retention, and to prevent skill decay [2-5]. However, there is little consensus on the optimal timing for simulation to prevent skill decay. Studies suggest that technical skills deteriorate as soon as two months after training if not used or practiced frequently [6-7]. Despite the evidence of rapid skill decay, there is often no monitoring of competence after initial learning [8]. This becomes particularly important in high stakes, low-frequency emergency procedures such as cardiopulmonary resuscitation (CPR). If providers are not regularly using these skills, they may be performing at a suboptimal competence level.

 Strategies using simulation to counteract skill decay are reported in the literature with terminology including “boosters,” “refreshers,” “just-in-time,” and “low-dose/high-frequency (LDHF)” sessions [1-4, 9]. This terminology is frequently overlapping, with an unclear definition of what strategies are most effective at different stages of skill decay. Furthermore, there is no standard terminology to describe types of training methodology used in simulation-based training. In this review, we will examine simulation-based training strategies and terminologies described in the literature and propose a framework for simulation strategies to achieve, maintain, or regain competency in clinical practice.

## Review

Simulation-based training strategies

Multiple simulation-based strategies have been described in the literature. These methods can be applied to different scenarios based on the learner’s baseline skill level, intervals of training, and the type of skill being acquired. An understanding of these strategies helps to delineate how they contribute to our framework for supporting the acquisition and maintenance of technical skills using simulation. We will describe four different strategies: deliberate practice, simulation-based mastery learning (SBML), just-in-time training, and LDHF training. 

Deliberate Practice

Anders Ericsson coined the term “deliberate practice” to describe a regimen of effortful activity designed to optimize improvements in the acquisition of expert performance [10-12]. The primary features of deliberate practice include motivated learners, well-defined learning objectives or tasks, focused and repetitive practice, precise measurements of performance, and informative feedback concerning performance [11]. Deliberate practice is an effective method for improving performance [13-14]. Deliberate practice has been shown to improve performance in advanced cardiac life support skills with little decay after three months [13]. It has also been shown to improve neonatal life support skills with three brief simulations spaced two to three months apart [14]. Deliberate practice has a role during initial training to help learners acquire competence in specific technical skills and in the prevention of skill decay. 

Simulation-Based Mastery Learning

Simulation-based mastery learning is a rigorous method to achieve a predefined performance standard through simulation-based deliberate practice until a ”mastery-level” standard is reached [5, 15]. The focus of SBML is for learners to achieve a high level of competency, and timing of training is determined by the learners’ ability to demonstrate mastery standard in the simulated environment [15]. Retention of mastery-level performance has a variable duration reported in the literature. One study reported a significant decline in pediatric resuscitation skills at four months after a single one to two hour session [8] while others showed retained intensive care skills for up to 12 months after a three-day boot camp [15] and maintained advanced cardiac resuscitation skills at 14 months after four, two-hour sessions [16]. Based on these findings, a dose-response relationship between the simulation intervention and the duration of the learning effect has been suggested such that the duration of skill retention is related to the intensity and duration of the initial simulation based intervention [5]. 

Just-In-Time Training

“Just-in-time” (JIT) training is a term used in the literature to define training sessions conducted immediately prior to performing a procedure on a patient [3]. Thus, it provides the learner with the ability to refresh skills on demand. This method of training has been shown to improve CPR quality [17] and frequent JIT refreshers resulted in a shorter amount of time to achieve CPR proficiency [3]. JIT training has also been used in procedural training such as lumbar punctures [18] and intubations [19]; however, some studies show JIT training has not been successful in increasing success in these procedures in trainees. This may be related to the baseline skill level at the time of JIT training. JIT training will not increase the skill, simply return the learner to a previously achieved competency level. Since JIT training occurs at the time that a procedure will be performed, this type of training may not prevent skill decay in procedures that the learner is not encountering in his or her clinical practice. ​​​​​​​

Low-Dose, High-Frequency Training

 Low-dose high-frequency training is characterized by short and targeted training sessions that are performed frequently in the workplace setting in order to maintain an adequate level of competence. In contrast to JIT training, where training occurs just prior to performing the procedure clinically, LDHF training may occur at any time. LDHF training can be performed at frequent and regular intervals in order to pre-emptively boost skills prior to decay, thus maximizing retention of skills. One study performed in Tanzania compared infant mortality and neonatal resuscitation interventions by providers who had gone through the “Helping Babies Breathe Program” before and after instituting weekly three- to five-minute training sessions using a simulator. The study found improved responsiveness in the initial steps of neonatal resuscitation, decreased need for bag mask ventilation, and an overall decrease in 24 hour mortality [20]. Regular CPR training for providers in a Pediatric Intensive Care Unit is another example of the effectiveness of LDHF training [3].

Training terminology

Initial Training

Initial training is when a skill is learned for the first time. Initial training is aimed at “novice” learners, as described by Dreyfus, with the goal to develop competency and reach a satisfactory level of performance [11]. This occurs in phases as the learner progresses through the Dreyfus levels. The sequential progression of an individual through these levels can be achieved through initial training using simulation-based deliberate practice and mastery learning, and then ongoing supervised clinical practice. ​​​​​​​

Maintenance Training

**Figure 1:**
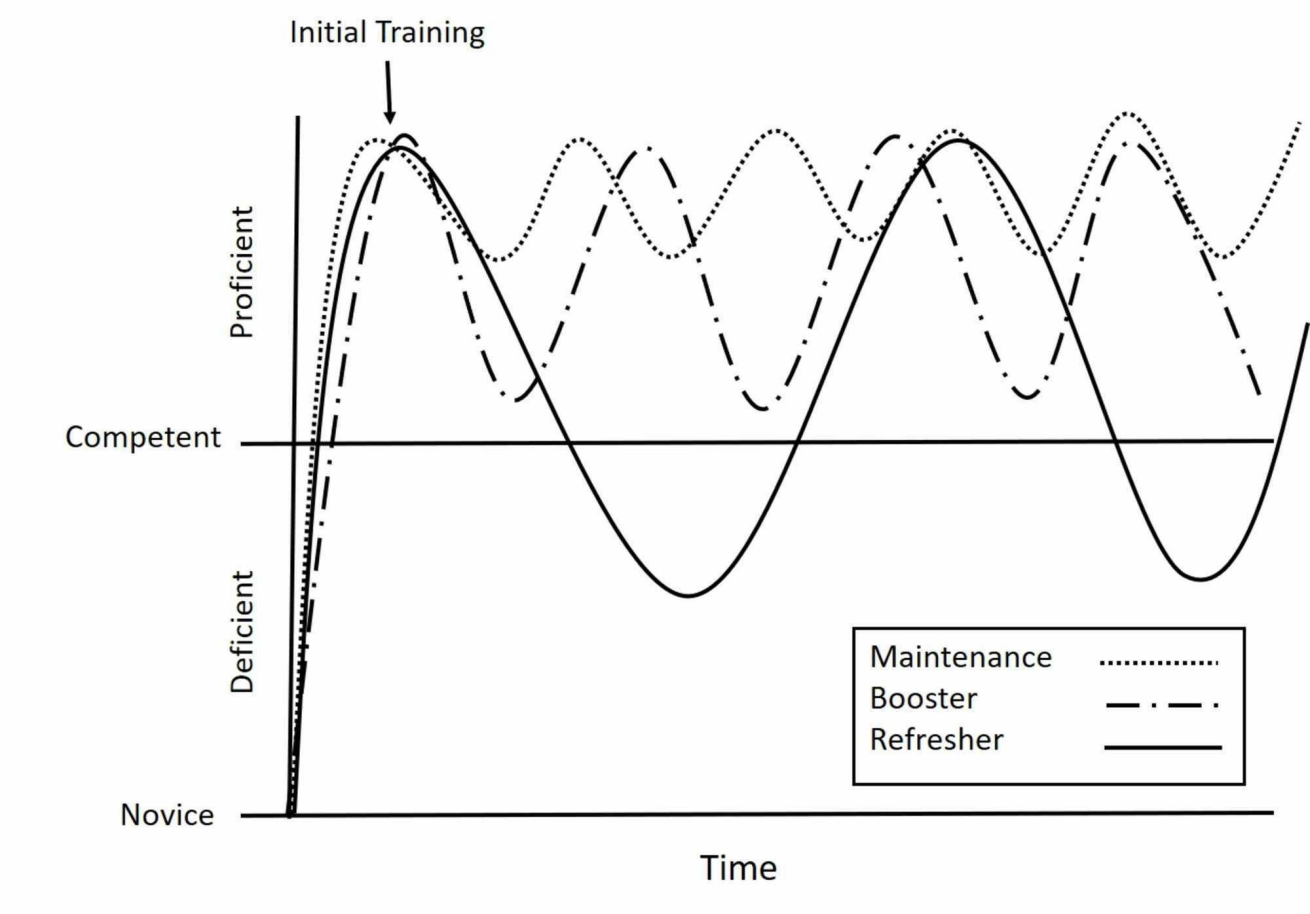
Maintenance, booster, and refreshers. This schematic demonstrates maintenance, booster, and refresher training with respect to skill decay over time. Maintenance involves high-frequency, but low-dose training sessions provided on an ongoing basis to maintain proficiency without losing competency. Booster training is used when proficiency has started to wane due to lack of use. The frequency of booster training is less than that of maintenance but with higher intensity, as is reflected in the greater amplitude of the curve. Refresher training becomes relevant when competency has diminished to unsatisfactory levels. This type of training requires a higher intensity than either maintenance or booster training.

Maintenance training is needed once competency is achieved through initial training. Maintenance training prevents skill deterioration and maintains a high level of competency over time. As demonstrated in Figure 1, maintenance involves high frequency, but low-dose training sessions provided on an ongoing basis. Maintenance-training strategies are well described in the literature. Sutton et al. used LDHF training to improve CPR skill retention over a six month period [4]. Bender et al. demonstrated neonatal resuscitation procedural and teamwork skills to be enhanced at 15 months after initial training with a half-day booster session nine months after initial training [9]. Rolling refreshers are another type of maintenance training. Rolling refreshers are a type of LDHF training provided at the bedside, and typically involves a rolling cart that is moved sequentially from bedside to bedside in order to train a number of clinical providers over a short period of time without separating the learners from clinical care [3].​​​​​​​

Booster Training

While maintenance of skills is ideal, it is not always feasible due to time, scheduling, and the clinical exposure of the learners. Booster training is used when competency has started to wane due to lack of use. The frequency of booster training is less than that of maintenance but with higher intensity, as is reflected in the greater amplitude of the curve in Figure 1. The higher intensity is necessary to achieve the desired competency level in the setting of skill decay. JIT training is an appropriate training strategy for booster training. In such a situation a provider who has not performed a skill for a prolonged period participates in a fairly intensive JIT training session before performing the procedure on a patient. Such JIT training ”oosts” skills back to an acceptable level at the time performance needs to be the best. ​​​​​​​

Refresher Training

Refresher training has been defined in the literature as an intervention that “aims to re-establish a specific skill level that was acquired at the end of initial training, which should be re-established after a period of non-use in which recall of the skill was not required" [1]. Thus, as Figure 1 demonstrates, refresher training becomes relevant when competency has diminished to unsatisfactory levels. This type of training requires a higher intensity than either maintenance or booster training. Traditionally, refresher training sessions are provided as part of life support courses such as Neonatal Resuscitation Program (NRP), Pediatric Advanced Life Support (PALS), and Advanced Cardiac Life Support (ACLS) which providers attend every two years. By relying on refresher training, rather than maintenance or booster training, providers run the risk of having inadequate skills to perform a life-saving intervention if faced with the situation prior to the refresher training. Because skills may have degraded significantly at the time of refresher training, deliberate practice is used in order to re-establish skills. 

A framework for supporting the acquisition and maintenance of technical skills using simulation

A framework to clarify the terminology around training using simulation for the acquisition and maintenance of technical skills and guidance on potential educational strategies is outlined in Table 1. This framework delineates four types of training: initial, maintenance, booster, and refresher. The framework differentiates these training types based on the learner competency at the time of the training, as well as the frequency and intensity of the training. Learner competency at the time of training can be described using the Dreyfus model of skill acquisition. Dreyfus describes the first stage as the “novice,” defined as learners who have an incomplete understanding, approach tasks mechanistically, and need supervision to complete them [21]. Through training, the learner advances from novice to “advanced beginner” as they are exposed to new situations and develop a working understanding but are only able to complete simple tasks [21]. While novice learners may achieve the level advanced beginner through training and deliberate practice, “competency” can only be achieved through clinical experience. Only through clinical experience, can a clinician acquire the ability to understand context and use judgment for troubleshooting more complex scenarios. Dreyfus defines competency as when the individual has a strong background understanding of the task and is able to work independently to an acceptable standard [21]. Once competency has been achieved, learners may achieve proficiency and expert level through ongoing clinical practice. “Proficiency” is only achieved with experience such that the learner has a deeper understanding, sees actions holistically, and can achieve a high standard of performance routinely. “Expert” performance is defined by Dreyfus, as the individual with vast experience in a variety of situations such that they are able to respond intuitively, achieving excellence with ease [21]. 

**Table 1:** A framework for supporting competency using simulation.

Type of training	Competency level at time of training	Frequency of training	Intensity of training	Simulation-based training strategies
Initial training	Not established	Once	Highest	Simulation-based mastery learning
Maintenance training	High	More frequent	Low	Low-dose, high-frequency or rolling refreshers
Booster training	Waning	Less frequent	High	Just-in-time training
Refresher training	Lost	As needed	Very high	Deliberate practice

 

Training intervals and training priorities

Various factors determine retention of competency after initial training. These factors include the baseline competency level of the learner, clinical exposure and frequency of skill use, and method of initial training. For example, one study demonstrated that providers with more experience had increased post-training retention in performing pediatric and neonatal intubation compared to novice providers [22]. Pusic et al. also described the variability in competency levels of learners in their demonstration of the heterogeneity in the individual learning curves of resident physicians practicing radiograph interpretation [23]. This heterogeneity in skill retention is a major challenge in being able to prescribe recommendations on standardized training intervals. Ideally, training intervals would be individualized and determined by learner characteristics and implications of the loss of skill.

Two methods of competency determination include time-based and skill-based. Time-based competency determination relies on an average time of skill decay for a specific provider group and uses the average time of skill decay to inform the type of training interval. An example of a time-based training schedule is providing maintenance training in intubation every six months to all intensive care physicians, regardless of their level of skill or clinical experience in the preceding six months. Skill-based competency determination requires a test of competency to determine which providers require maintenance training. Such tests can be done on a scheduled basis, or on an as-needed basis before a skill is used clinically. A hybrid approach may involve regular maintenance sessions scheduled at a set interval with the inclusion of a competency test. Such a method ensures that all providers have access to maintenance training while allowing providers that pass the competency assessment to opt out of training and return at the next scheduled time. 

The implication of the loss of skill should be used to prioritize the skills being taught. Loss of competency of low volume, high-risk skills such as life-saving skills and emergency procedures could have significant negative consequences. Thus, maintenance and booster training are more appropriate than refresher training for these types of skills. For nonemergent life-saving skills, which are also important, the need for ongoing, continuous, competency is not as vital, making them more suitable for refresher training. Examples include placement of chest tubes, lumbar punctures, and exchange transfusions. 

## Conclusions

Traditional medical education and the current life support course training processes rely on refreshers to supplement initial learning. With the framework we propose, competency of certain skills, particularly high-risk, lifesaving skills, should be maintained with more frequent training at lower levels of intensity. Retraining intervals can be determined using time-based or skill-based methods. Using a framework to mitigate skill decay and support competency, will encourage an increased emphasis in medical education towards maintenance and booster training in order to preserve skills before competency is lost. We hope that the use of a standardized nomenclature will also help provide clarity for future research opportunities in the best methods for preservation of skill. 
